# Stable Organic Passivated Carbon Nanotube–Silicon Solar Cells with an Efficiency of 22%

**DOI:** 10.1002/advs.202102027

**Published:** 2021-09-02

**Authors:** Jun Yan, Cuili Zhang, Han Li, Xueliang Yang, Lu Wan, Feng Li, Kaifu Qiu, Jianxin Guo, Weiyuan Duan, Andreas Lambertz, Wanbing Lu, Dengyuan Song, Kaining Ding, Benjamin S. Flavel, Jianhui Chen

**Affiliations:** ^1^ Hebei Key Lab of Optic‐Electronic Information and Materials College of Physics Science and Technology Hebei University Baoding 071002 China; ^2^ Institute of Nanotechnology Karlsruhe Institute of Technology 76344 Eggenstein‐Leopoldshafen Germany; ^3^ State Key Laboratory of Photovoltaic Materials & Technology Yingli Green Energy Holding Co., Ltd. Baoding 071051 China; ^4^ IEK5‐Photovoltaics Forschungszentrum Jülich Wilhelm‐Johnen‐Strasse 52425 Jülich Germany

**Keywords:** organic passivation, photovoltaics, silicon solar cells, single‐walled carbon nanotube (SWCNT)

## Abstract

The organic passivated carbon nanotube (CNT)/silicon (Si) solar cell is a new type of low‐cost, high‐efficiency solar cell, with challenges concerning the stability of the organic layer used for passivation. In this work, the stability of the organic layer is studied with respect to the internal and external (humidity) water content and additionally long‐term stability for low moisture environments. It is found that the organic passivated CNT/Si complex interface is not stable, despite both the organic passivation layer and CNTs being stable on their own and is due to the CNTs providing an additional path for water molecules to the interface. With the use of a simple encapsulation, a record power conversion efficiency of 22% is achieved and a stable photovoltaic performance is demonstrated. This work provides a new direction for the development of high‐performance/low‐cost photovoltaics in the future and will stimulate the use of nanotubes materials for solar cells applications.

## Graphical abstract

A new type of solar cell, based on the conductive passivating contacts being consisted of a CNT:Nafion mixed system, provides a promising new avenue for low‐cost, high‐efficiency photovoltaic devices. Compared with the current best a‐Si:H(i/p^+^) passivating contact cells (silicon heterojunction), parts of heavy vacuum equipment are omitted while a stability trend and record high efficiency of >22% are still retained.

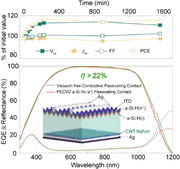

## 1 Introduction

Silicon solar cells are clearly the most successful and prolific photovoltaic(PV) technology of our time. Ninety seven percent of the global PVs market can be accredited to silicon based cells,^[^ ^1^, ^2^ ^]^ recently industrialized solar cell architectures such as the passivated emitter and rear cell (PERC) have reached power conversion efficiencies (PCEs) of ≈ 23%,^[^ ^3^ ^]^ and new generation scaled silicon heterojunction cells have pushed past 24% on industrial production lines^[^ ^4^ ^]^ with interdigitated back contacts reporting 26.7% in the laboratory.^[^ ^5^ ^]^ However, these impressive PCEs are coupled to increasingly complex device designs and these have correspondingly high manufacturing costs. The dominant feature for the recent high‐efficiencies is the “passivating‐contact,” which consists of a carrier‐selective contact (phosphorous, n^+^ or boron, p^+^)^[^ ^6^ ^]^ overlaid by a passivation layer (SiO_2_ or intrinsic hydrogenated amorphous Si (a‐Si:H(i)).^[^ ^7^, ^8^ ^]^ These silicon layers are energy intensive to fabricate, require high vacuum processes, and are deposited serially on the production line. Therefore, in order to reduce the levelized cost of production, new and simplified approaches are urgently required. For example, water‐based dye‐sensitized solar cells and perovskites solar cells have attracted the great attention as a cheap and efficient alternative.^[^ ^9^, ^10^, ^11^, ^12^, ^13^, ^14^ ^]^

In this direction, hybrid materials are being developed that can passivate interfacial electron traps whilst also being carrier selective. These materials have been named “conductive passivating contacts (c‐PCs)” and one such approach is to disperse carrier selective nanoparticles in an organic passivation agent to form an ink that can be spin coated in a single step and at room temperature.^[^ ^15^ ^]^ On their own, organic thin films with the sulfonic acid group (‐SO_3_H), such as polystyrenesulfonate,^[^ ^16^ ^]^ poly(2‐acrylamido‐2‐methylpropanesulfonic acid), polystyrene*‐block‐*poly(ethylene‐ran‐butylene)*‐block‐*polystyrene‐sulfonated‐cross‐linkable,^[^ ^17^ ^]^ and Nafion have all been shown to effectively passivate interfacial trap states on silicon by the electrochemical grafting of —SO_3_H onto Si surface dangling bonds to form Si suboxides at the polymer/Si interface.^[^ ^18^ ^]^ More specifically, minority carrier lifetimes of 9.6–28.6 ms have been reported for organic passivation and these rival industry standard hydrogenated amorphous Si (a‐Si:H) or SiO_2_ film‐passivation schemes.^[^ ^19^, ^20^ ^]^ For c‐PCs such as PEDOT:Nafion and CNT:Nafon, high PCEs of 18.8% and 21.4% for industrial p‐type polycrystalline and n‐type monocrystalline silicon solar cells, respectively, have been demonstrated.^[^ ^15^, ^21^ ^]^ In terms of simplicity and the resultant PCE, this approach compares favorably with the recently industrialized PERC but allowed for the use of n‐type silicon to form a new heterojunction and was predicted to be compatible with other nanoparticles including flakes of graphene,^[^ ^22^, ^23^, ^24^, ^25^ ^]^ MXenes,^[^ ^26^ ^]^ or transition metal dichalcogenides.^[^ ^27^ ^]^

From the CNT‐based PV device perspective, the c‐PC approach provided a breakthrough in performance for CNT‐Si solar cells, but also for the area (245.71 cm^2^) achieved.^[^ ^21^ ^]^ However, the downside of the approach was the long term stability of the CNT:Nafion composite layer. Although CNTs have excellent stability toward degradation in ambient, humid, hot, or ultraviolet radiation conditions,^[^ ^28^, ^29^, ^30^, ^31^ ^]^ the hygroscopic nature of Nafion is known to lead to performance reductions^[^ ^32^ ^]^ and must be addressed. Primarily, these issues are related to reductions in the open circuit voltage (*V*_oc_) from degradation of the Nafion based passivation. In similar work by Qian et al.,^[^ ^33^ ^]^ who also combined Nafion and CNTs, device stability was reported to be stable but this is likely due to the lower PCE/*V*_oc_ of 14.4%/549 mV. In other work we have shown that the stability of the passivation effect afforded by a ‐SO_3_H based organic thin film alone can be increased to 430 days with the use of an ALD‐Al_2_O_3_ encapsulation layer, but such a layer is incompatible with c‐PC solar cells because of the insulating property of Al_2_O_3_. Nevertheless, it does suggest that organic passivation strategies alone can become stable via encapsulation.^[^ ^15^ ^]^ For a solar cell, the addition of CNTs is required to provide the conductive and carrier selective component of the c‐PC and here the method of their inclusion appears to be important. When Nafion and the CNTs were combined together into a single ink, the high PCE (21%) of our devices was found to be stable for only 20 min, whereas when a layer of CNTs was first laminated onto the silicon wafer and then coated with Nafion in a second step, performance was found to be stable for 2 days.^[^ ^34^ ^]^ Clearly, a mixed system that can be deposited in a single step is more attractive for industry if stability can be improved. In this work, the stability of CNT:Nafion mixed systems are studied, both by varying the content of water within the film, but also the humidity of the external environment. Device compatible encapsulation is then employed to demonstrate a stability trend and record high PCE of 22%. In addition, a high fill factor (FF) of 83.4% and external quantum efficiency (EQE) which can rival the current a‐Si:H(i/p^+^) passivating contact, are obtained, revealing the potential advantage of the CNT‐organic c‐PC technology.

## 2 Results and Discussion

**Figure 1:**
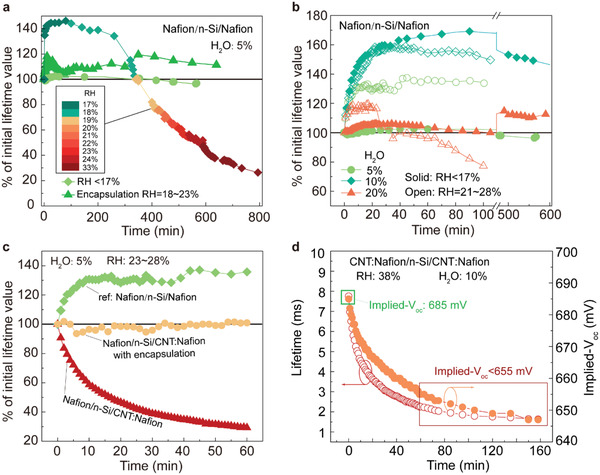
Lifetime determined from transient photoconductance decay measurements of a) a Nafion film with increasing relative humidity (RH), b) varied internal water content (5–20%) in dry and humid environments, and c) in combination with carbon nanotubes. d) Implied *V*_oc_ calculated from at peak passivation and after 80 min.

Using the technique of the quasi steady‐state and transient photoconductance decay (PCD), the minority carrier lifetime (*τ*_eff_) for silicon wafers (400 µm‐thick n‐type (100)‐oriented slightly doped, with the resistivity of 3–5 kΩ•cm) coated symmetrically with a Nafion film alone were measured and are shown in **Figure** **1**a. Nafion films were spin cast from an alcohol/water mix (2.94 wt% Nafion/92.06 wt% aliphatic alcohols/5 wt% H_2_O) and the relative humidity (RH) was increased from 17–33% over a period of 800 min. To aid comparison, the initial value of *τ*_eff_ (4–20.6 ms), measured immediately after coating, was used for normalization. The initial increase in lifetime (up to ≈ 146%, 20.6 ms) is related to the electrochemical passivation effect of Nafion. This effect is enhanced by O_2_ in the atmosphere (the basic principle of the O_2_‐enhanced passivation can be found elsewhere^[^ ^32^ ^]^) but the ingress of small quantities of water into the film has a negative effect and *τ*_eff_ was reduced to ≈ 26% for a RH of 33%. In contrast, when the RH was held at 17% or when an Al_2_O_3_ encapsulation layer was used, *τ*_eff_ was found to be stable for 10 h. Figure 1b shows the normalized *τ*_eff_ evolution for Si wafers passivated by the Nafion thin‐films with different water content (5–20%) in the solution to be spin coated. Once again, for a RH < 17% no obvious degradation in lifetime is observed for each of the films. However, for a RH > 17% and a water content of 20%, degradation of the lifetime begins to occur. The passivation effect of Nafion can be summarized in the following way: i) the passivation is degraded by the increase of the water within film and RH in the environment; ii) when the water content is < 20% and RH is < 17%, long term stability can be achieved. This is unlike conventional chemical passivation or field‐effect passivation mechanisms and depends on the electrochemical interaction between —SO_3_H and Si surface, according to the Equation (1),^[^ ^18^ ^]^

(1) $$\equiv \mathrm{Si}\cdot +X-{\mathrm{SO}}_{3}H\;\underset{e^{-}}{\overset{h^{+}}{⇌}}\mathrm{Si}-O-R^{\prime }$$

where X represents a normal constituent other than a sulfonic functional group, ‐SO_3_H, and R´ is the surplus O‐containing constituent in the X‐SO_3_H molecule. The water molecule invasion will contribute electrons to the Si surface via,

(2) $$2\mathrm{Si}-O-R^{\prime }+H_{2}O\to 2\left(X-{\mathrm{SO}}_{3}H\right)+\frac{1}{2}O_{2}+2e^{-}+2\equiv \mathrm{Si}\cdot$$

which favors the reverse reaction in Equation (1) and results in the degradation of passivation.

Figure 1c shows the normalized *τ*_eff_ evolution of the Si wafers passivated by a CNT:Nafion thin‐film spin cast from a solution with a water content of 5%. Samples with and without Al_2_O_3_ encapsulation at 23% RH are shown along with the first 60 min of the Nafion only trace in Figure 1b for reference. Unexpectedly, the addition of the CNTs leads to a decrease in the stability with reduction of ≈69% of the initial lifetime value already occurring within this short time period. This suggests that CNTs accelerate the ingress of water, most likely by increasing the porosity of the film but possibly also due to endohedral water transport to the interface.^[^ ^35^ ^]^ This physiochemical mechanism is sketched in Figure S1, Supporting Information. A MoO_3_ nanoparticle (≈ 5 nm) was used to fill the holes in the CNT:Nafion layer and stop the ingress of water and this lead to a slight increase in stability (Figure S2, Supporting Information), but a denser Al_2_O_3_ layer completely eliminated water, as shown in Figure 1c. Note that before and after the addition of MoO_3_ nanoparticles, large difference in the electrical conductivity (Table S1, Supporting Information) and work function (Figure S3, Supporting Information) were not observed. However, the use of an insulating Al_2_O_3_ layer for encapsulation prevents carrier transport when used in a back‐junction architecture and when full rear contact with the c‐PC layer is desired. For solar cells, a conductive encapsulation material is required.

**Figure 2:**
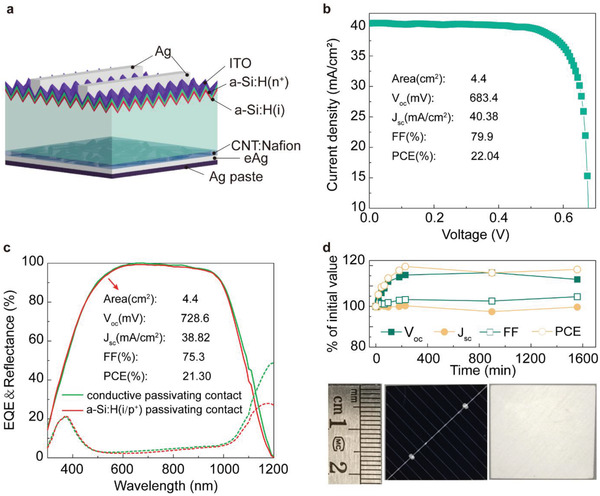
a) Schematic of the c‐PC based device. The eAg layer is an evaporated Ag layer as a back electrode. b) *J–V* curve of the champion solar cell device with performance values inset. c) The external quantum efficiency (EQE) measurements and reflectance spectra comparison between conductive passivating contact cell and a‐Si:H(i/p^+^) passivating contact cell with performance values inset. d) Stability of *V*_oc_, *J*_sc_, FF, and PCE over a period of 1600 min in ambient conditions. Note: the solar cells were not illuminated continuously during this time. Photographs of the completed devices showing the front and rear of the cell.

The technique of PCD allows for the implied *V*_oc_ to be calculated using the following equation:

(3) $$\mathrm{implied}-V_{\mathrm{oc}}=\frac{kT}{q}\;\ln\left(\frac{\Delta n\left(\Delta n+N_{D}\right)}{n_{i}^{2}}\right)$$

where Δ*n* is the excess carrier density, *n*_i_ is the intrinsic carrier concentration and *N*_D_ is the effective bulk donor concentration. Using this approach, the best possible *V*_oc_ that can be expected from a real solar cell can be predicted based on the peak passivation effect and before degradation of the lifetime. Figure 1d shows *τ*_eff_ and implied *V*_oc_ as a function of the time for an un‐encapsulated CNT:Nafion film with 10% water content. As shown earlier, a Nafion film with 5% water content has the least water and therefore the most stable passivation effect, but it also results in the thinnest films from spin coating. For the final use with CNTs in a solar cell, a 10% water Nafion film was the best compromise between the required thickness and passivation, see Figure S4 and Table S2, Supporting Information. Taking the initial peak value of 7.75 ms affords a high implied *V*_oc_ of 685 mV. Back junction solar cells were then built from such a CNT:Nafion film and a schematic of the complete device is shown in **Figure** **2**a. Such high‐quality passivation of a CNT:Nafion c‐PC layer can also be shown by spatially resolved PL measurements of the samples before and after passivation (Figure S5, Supporting Information). An electron‐selective passivation contact comprising n‐doped hydrogenated amorphous silicon stack thin film (a‐Si:H(i/n^+^)) with a transparent conductive ITO antireflection layer is used on the front side. A CNT:Nafion c‐PC layer is spin coated on the back side, followed by the evaporation of a thin Ag film (eAg). In this design, the CNT‐Si heterojunction is responsible for hole‐selective transport like a p‐n junction and the Nafion‐Si organic‐inorganic interface is responsible for the passivation of the interfacial electron traps. Encapsulation was performed with an electrical Ag paste and forms a dense coating on the eAg film. Figure 2b shows the illuminated *J–V* curve of the champion device. A record high PCE of 22.04% (Table S3, Supporting Information), a *V*_oc_ of 683.4 mV, a *J*_sc_ of 40.38 mA cm^−2^, and a FF of 79.9% were obtained. Figure 2c shows the EQE and reflectance curves. A p‐doped hydrogenated amorphous silicon stack thin film (a‐Si:H(i/p^+^)) passivating contact cell is shown as a reference to demonstrate the improvement of the infrared light management in the c‐PC cell, which has the benefit of rear surface chemical polishing and low refractive index of the back c‐PC layer^[^ ^23^ ^]^ in this kind of solar cell. Note that only *V*_oc_ needs to be further improved compared with the a‐Si:H(i/p^+^) passivating contact cell. As shown in Figure 2d PV performance values were found to be stable over a period of 1600 min. *V*_oc_ exhibits an increase in a short initial period, which is ascribed to the O_2_‐enhanced operating mechanism by the Nafion passivation.^[^ ^32^ ^]^

**Figure 3:**
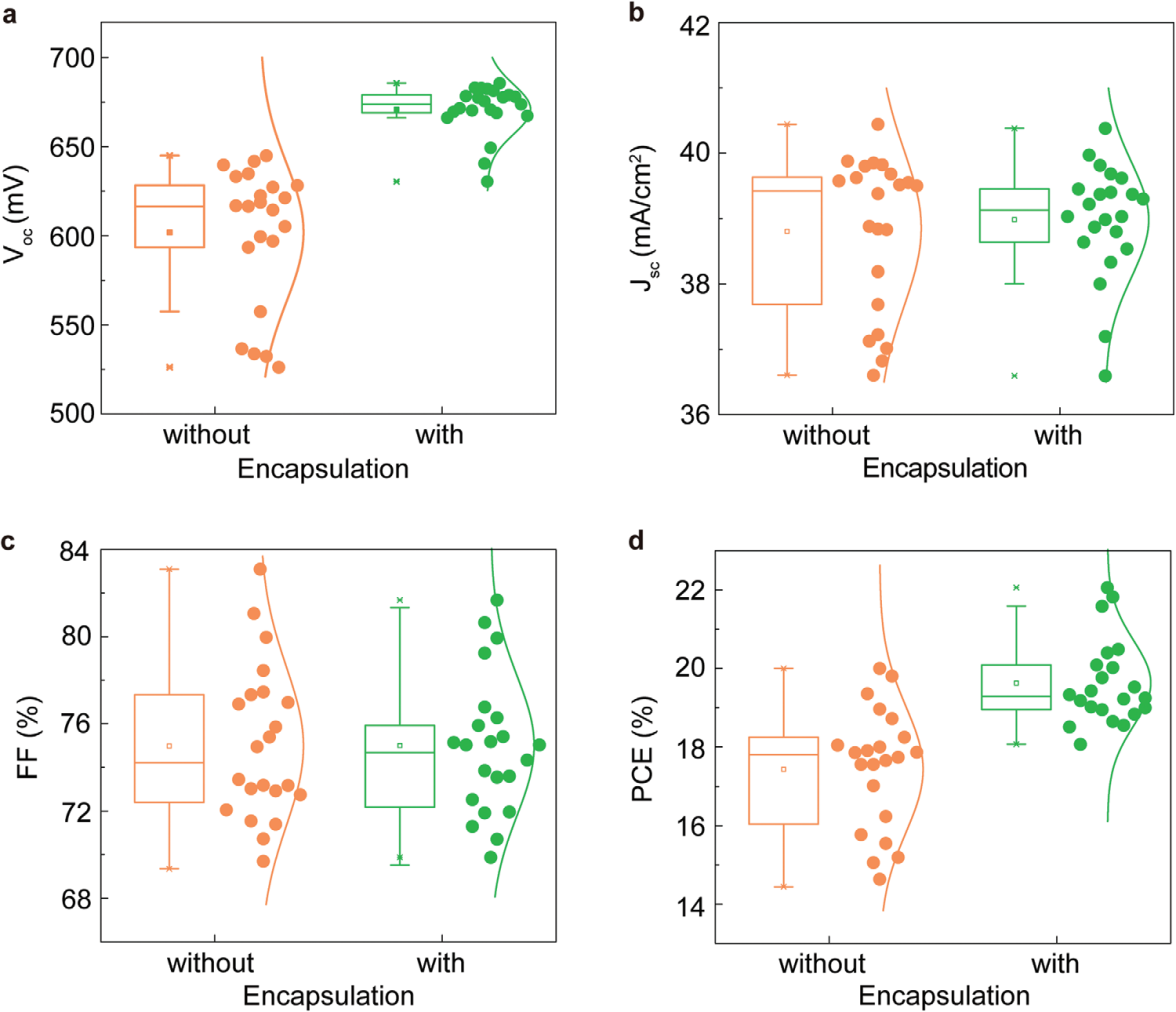
Comparison of the solar cell performance values a) *V*_oc_, b) *J*_sc_, c) FF, and d) PCE with and without encapsulation.

In **Figure** **3** the performance values for 44 devices are presented and a comparison is made between encapsulated and un‐encapsulated cells, Table S4, Supporting Information showed the PV parameters with the error analysis. In our laboratory, the process of CNT:Nafion thin film coating, metallization, and finally measurement requires at least 1 h of time, during which the solar cell is exposed to water. This results in a reduction in *V*_oc_ (683 mV) compared to the encapsulated devices (Figure 3a), but it compares well to the lower implied *V*_oc_ of < 655 mV in Figure 1d at 1 h. As it can be seen in Figure 3b,c, encapsulation generally does not affect the *J*_sc_ or FF, the former is due to the stable and consistent optical property on the front of devices and the later suggests that the electrical contact (i.e, CNT:Si) on the rear of the device is stable. The small variation in parameters can be seen and is attributed to the device stability and the preparation of the ink with the good dispersion. In fact, the FF can reach 83.4%, as shown in Figure 3c, which is unusually high and revels an advantage of the CNT‐based c‐PC technology compared other nanomaterial based passivating contact approaches. We attribute this result to the high mobility of the CNTs^[^ ^36^, ^37^ ^]^ and high‐quality passivation of Nafion. Figure 2c compares the EQE response of a‐Si:H(i/p^+^) passivating contact solar cell to ours. Both cells have the same geometry except for the back‐side c‐PC or a‐Si:H(i/p^+^) passivating contact. Both have a similar EQE, suggesting that the c‐PC can have the same level performance potential with the current best a‐Si:H(i/p^+^) passivating contact. Further work is now required to increase the *V*_oc_, of c‐PC based solar cells, but now with a proven environmental stability, the combination of nanomaterials with organic passivation layers appears to provide a promising new avenue for low‐cost and high‐PCE PV devices in the future.

## 3 Conclusion

The a‐Si:H passivating contact has been a great success to the PV community due to the super passivation of a‐Si:H(i) and good carrier‐selective transport properties of a a‐Si:H(p)/a‐Si:H(i)/n‐Si p‐i‐n junction. In this work, a single composite c‐PC layer with dual (carrier selective and passivating) has been shown to be long term stable with simple encapsulation. Following encapsulation, a new record in CNT:Si solar cell performance was obtained and a PCE of 22.04% reported. An EQE that is similar to industry standard passivated junctions also showed this approach is becoming competitive with existing technology. In the long term, because stability is limited by water and final solar cells are always encapsulated in a PV module, a c‐PC approach is highly attractive for the vacuum‐free, room temperature, low‐cost fabrication.

## 4 Experimental Section

### Solution and Thin Film Preparation

Nafion solutions with different water content (5%, 10%, and 20%) were obtained by mixing an original 20 wt% Nafion (Sigma Aldrich, 20 wt% in a mixture of lower aliphatic alcohols and 34% water) with ethanol (> 99.7 wt%). Simple magnetic stirring for 12 h was used to obtain the uniform precursor solution. The c‐PC ink was prepared by dispersing raw electric‐arc single‐wall CNT powder (XFS28, XFNano) with Nafion solution in the ratio of 1.8 mg mL^−1^ by shear force mixing for 3 h. The CNT:Nafion composite thin‐films were spin‐coated at 5000 rpm for 60 s. The two‐side coated silicon wafer was placed into the ALD chamber and 99% trimethylaluminum (Al (CH_3_)_3_, TMA) used as the aluminum source of ALD reaction with deionized (DI) water used as the oxidizer. High purity nitrogen was used as the carrier gas and to purge the chamber. A 20 ms pulse time for the TMA and DI Water was used and a 20 s purge time. The substrate temperature for deposition was 90 °C and the number of cycles was used to control the thickness. A cycle of 40 s, 45 times afforded an alumina film thickness of 10 nm, the thickness of alumina film was ≈10 nm.

### Solar Cells

Device fabrication involved several steps: 1) wet chemistry, including texturing and cleaning of wafer surface with the method found elsewhere;^[^ ^38^, ^39^ ^]^ 2) back surface polishing using a HNO_3_/HF/H_2_O (4:1:2) mixed solution, which was highly associated with the final CNT:Nafion film morphology and hence important to reproducibly achieve high performance; 3) the formation of the front electron‐selective passivating contact, antireflection layer, and metallization; 4) spin coating of a c‐PC CNT:Nafion thin‐film on the relatively flat back side using a CNT/Nafion ink under N_2_ atmosphere at room temperature. 5) thermal evaporation of a 300 nm full contact Ag electrode on CNT:Nafion thin film, coated by a Ag paste (SPI Supplies, 05001) was applied for encapsulation. On the front side, a‐Si:H(n^+^)/a‐Si:H stack layer was deposited using a plasma‐enhanced chemical vapor deposited (PECVD) equipment on the textured front surface of n‐type wafers, and then an ITO layer was deposited by magnetron sputtering on the top of the a‐Si:H(n^+^).^[^ ^40^, ^41^ ^]^ Subsequent metallization of front surface was performed by screen‐printing with an Ag paste, followed by a curing process at 170 °C for 40 min. This reproducibility of the final device performances was enabled by the spin‐coating method, stable back surface wet chemistry, the preparation of the ink with the accurate CNT‐Nafion ratio, and the water resistance encapsulation.

### Characterization

The lifetime was measured by spin‐coating CNT:Nafion films on both sides of floated‐zone Si wafer (400 µm, n‐type, 3–5 kΩ•cm) double‐sided mirror‐polished surfaces with an Sinton WCT‐120 instrument. A “transparent plastic box” with gas switches that could be directly mounted onto the WCT‐120 measurement stage to provide a stable dry air atmosphere.^[^ ^32^ ^]^ The performances of the solar cells were characterized by current density‐voltage (*J*–*V*) measurements under standard test conditions (AM 1.5, 100 mW·cm^−2^ and 25 °C) and the EQE (R3011, Enlitech).

## Conflict of Interest

The authors declare no conflict of interest.

## Supporting information

Supporting Information

## Data Availability

Research data are not shared.
