# Supplementary material for: Stable Organic Passivated Carbon Nanotube–Silicon Solar Cells with an Efficiency of 22%
Source: Adv Sci (Weinh). 2021 Sep 2;8(20):2102027. doi: 10.1002/advs.202102027 (PMC8529485; doi:10.1002/advs.202102027)
Supplement: Supplementary file 1 — Supporting Information [file ADVS-8-2102027-s001.pdf]

© 2021 Wiley-VCH GmbH

ADVANCED SCIENCE
Open Access

Supporting Information

for *Adv. Sci.,* DOI: 10.1002/advs.202102027

Stable Organic Passivated Carbon Nanotube-Silicon Solar Cells with an Efficiency of 22%

Jun Yan, Cuili Zhang, Han Li, Xueliang Yang, Lu Wan, Feng Li, Kaifu Qiu, Jianxin Guo, Weiyuan Duan, Andreas Lambertz, Wanbing Lu, Dengyuan Song, Kaining Ding, Benjamin S. Flavel and Jianhui Chen**

Supporting Information

Stable Organic Passivated Carbon Nanotube-Silicon Solar Cells with an Efficiency of 22%

Jun Yan[1], Cuili Zhang[1], Han Li[2], Xueliang Yang[3], Lu Wan[1], Feng Li[3], Kaifu Qiu[4], Jianxin Guo[1], Weiyuan Duan[4], Andreas Lambertz[4], Wanbing Lu[1], Dengyuan Song[1, 3], Kaining Ding[4], Benjamin S. Flavel[2] and Jianhui Chen[1,2*]*

Corresponding author.

E-mail address: chenjianhui@hbu.edu.cn (J. Chen); benjamin.flavel@kit.edu (B. S. Flavel)

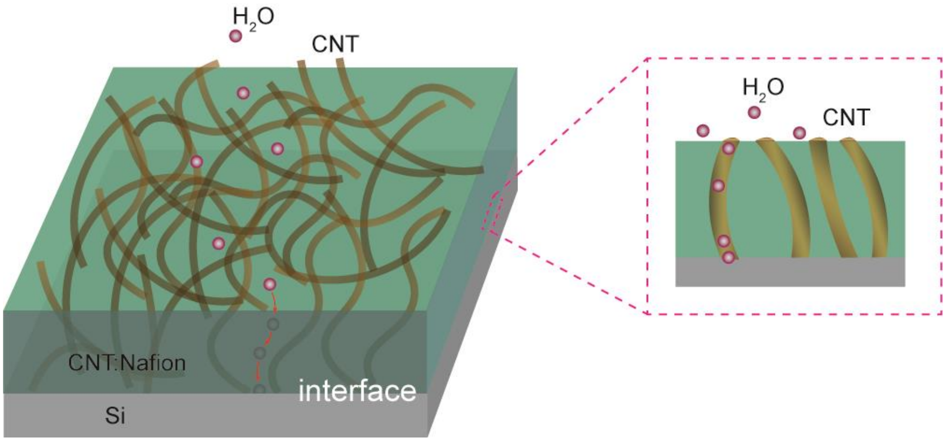

Figure S1. Physiochemical mechanism of the CNT/Nafion and Si interfaces.

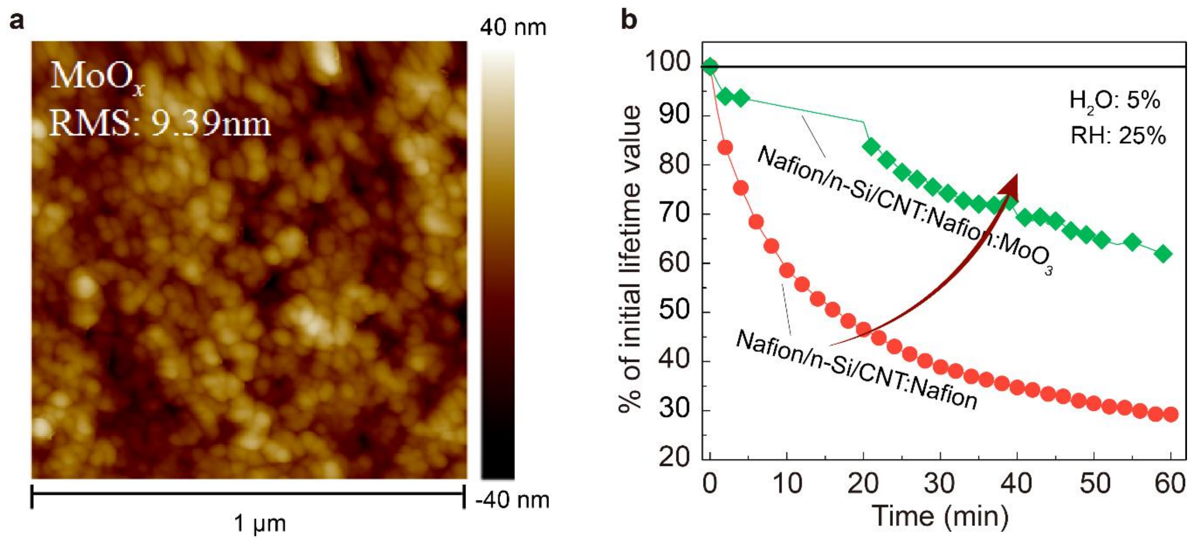

Figure S2. (a) AFM image of MoO_x nanoparticles; (b) the normalised lifetime afforded by a CNT:Nafion film with and without MoO_x nanoparticle filling.

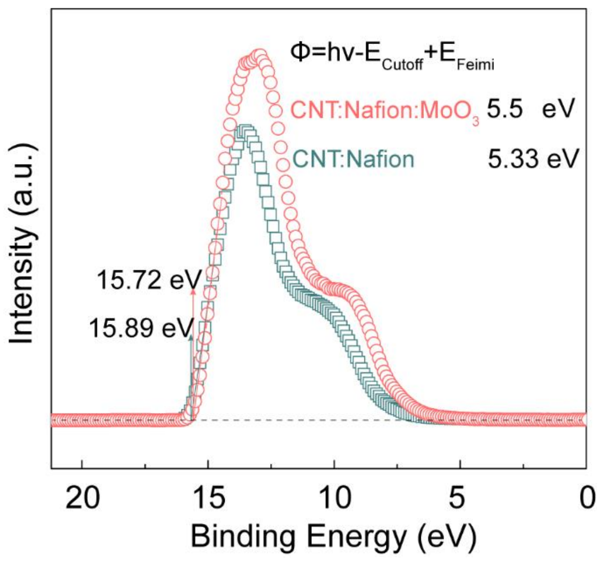

Figure S3. Ultraviolet photoelectron spectroscopy of CNT:Nafion and CNT:Nafion:MoO_3 films.

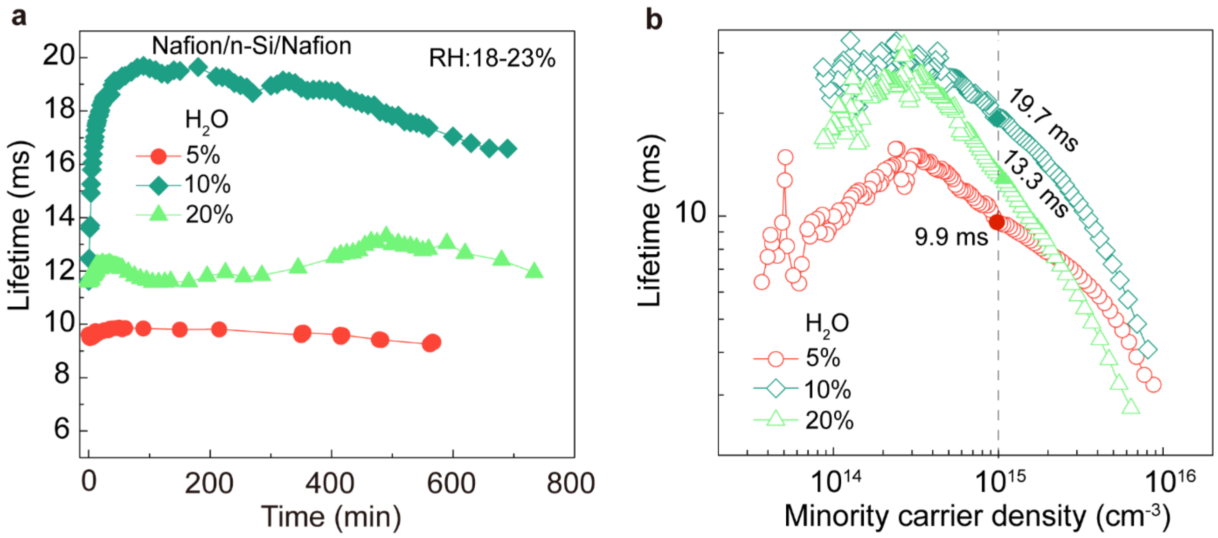

Figure S4. (a) Lifetime values of Nafion films with 5, 10 and 20% water content at a relative humidity of 18-23% and (b) the peak lifetime curves.

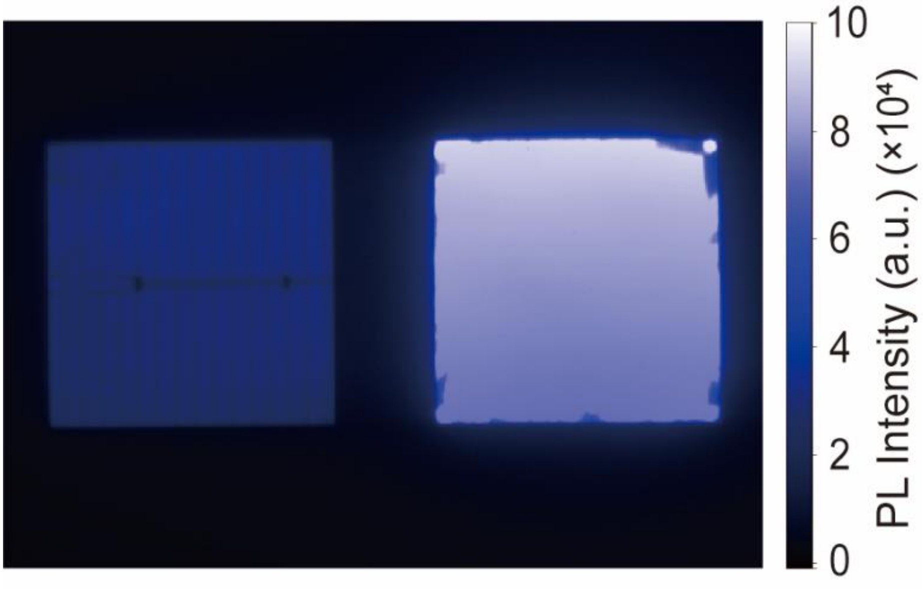

Figure S5.PL measurements of the Ag/ITO/a-Si:H(n^+)/a-Si:H(i)/n-Si test geometry with and without the back c-PC layer.

Table S1. The electrical conductivity of CNT:Nafion and CNT:Nafion:MoO_3 films.

Materials	Conductivity (S/cm)
CNT:Nafion	9.2×10^{-2}
CNT:Nafion:MoO_3	5.9×10^{-2}

Table S2. Thickness of the Nafion thin filmwith different water content.

Water content (wt.%)	Thickness (nm)	Lifetime (ms)
5%	109±22	8.9
10%	252±44	13.1
20%	722±29	7.4

Table S3. Reported efficiencies by publication year.

**Year**	***PCE*(%)**	***FF* (%)**	**V_{oc} (%)**	**J_{sc}(mA/cm^2)**	**Area(cm^2)**	**Affiliation**	**Device geometry**
2007	1.4	19	500	13.8	0.49	TsinghuaUniversity[1]	
2008	7.4	53	540	26	0.49	TsinghuaUniversity[2]	
2009	4.5	49	350	26.5	0.25	ArkansasUniversity[3]	
2010	10.9	79	550	25	0.08	Florida University[4]	
2011	10.9	67.6	560	29	0.09	TsinghuaUniversity[5]	
2012	15.1	77	610	32	0.15	Peking University[6]	
2013	11.5	74	530	29.3	0.09	Yale University[7]	
2014	10.8	69	510	31	0.49	Yale University[8]	
2015	17	78	590	36.6	0.00785	KyotoUniversity[9]	
2016	10.11	51.97	630	25.32	2.15	Peking University[10]	
2017	14.09	72.3	540	36.1	0.09	Kyoto University[11]	Window-like geometry
2019	14.8	71.2	618	33.7	0.09	Peking University[12]	
2019	14.4	71.2	549	36.7	0.09	Kyoto University[13]	
2019	17.2	76.3	659	32.3	1	KIT[14]	
2020	18.9	77.2	631	38.8	3	KIT/HBU[15]	
2020	21.4	82	654	39.9	4.8	HBU/KIT [16]	
2020	20.1	78.9	646	39.5	245.71	HBU/KIT[16]	Industrial geometry
2021	22	79.9	683.4	40.38	4.4	HBU/KIT (This work)	

(KIT:Karlsruhe Institute of Technology; HBU: Hebei University)

Table S4. PV parameters of carbon nanotube-silicon solar cells with and without encapsulation.

Encapsulation	V_{oc} (mV)	J_{sc}(mA/cm^2)	FF (%)	PCE (%)
W/O	601.3±38.9	38.85±1.2	75.3±3.6	17.6±1.6
W	670.7±14.0	38.98±0.9	74.9±3.2	19.6±1.1

References

[1] J. Wei, Y. Jia, Q. Shu, Z. Gu, K. Wang, D. Zhuang, G. Zhang, Z. Wang, J. Luo, A. Cao, D. Wu, *Nano Lett*. **2007**, 7, 2317.

[2]Y. Jia, J. Wei, K. Wang, A. Cao, Q. Shu, X. Gui, Y. Zhu, D. Zhuang, G. Zhang, B. Ma, L. Wang, W. Liu, Z. Wang, J. Luo, D. Wu, *Adv. Mater.***2008**, 20, 4594.

[3]Z. Li, V. P. Kunets, V.Saini, Y. Xu, E. Dervishi, G. J. Salamo, A. R. Birs, A. S. Biris, *ACS Nano*,**2009**, 3,1407.

[4]P. Wadhwa, B. Liu, M. A. McCarthy, Z. Wu, A. G. Rinzler, *Nano Lett.***2010**, 10, 5001.

[5]Y. Jia, P. Li, X. Gui, J. Wei, K. Wang, H. Zhu, D. Wu, L. Zhang, A. Cao, Y. Xu, *Appl. Phys.Lett*. **2011**, 98, 133115.

[6]E. Shi, L. Zhang, Z. Li, P. Li, Y. Shang, Y. Jia, J. Wei, K. Wang, H. Zhu, D. Wu, S. Zhang, A. Cao, *Sci. Rep.***2012**, 2, 884.

[7] X. Li, Y. Jung, K. Sakimoto, T.-H. Goh, M. A. Reed, A. D. Taylor, *Energy Environ.Sci.***2013**, 6, 879.

[8] X. Li, Y. Jung, J.-S. Huang, T. Goh, A. D. Taylor, *Adv. Energy Mater.***2014**, 4, 1400186.

[9] F. Wang, D. Kozawa, Y. Miyauchi, K. Hiraoka, S. Mouri, Y. Ohno, K. Matsuda, *Nature commun.***2015**, 6, 6305.

[10] W. Xu, S. Wu, X. Li, M. Zou, L. Yang, Z. Zhang, J. Wei, S. Hu, Y. Li, A. Cao, *Adv. Energy Mater.***2016**, 6, 1600095.

[11] K. Cui, Y. Qian, I. Jeon, A. Anisimov, Y. Matsuo, E. I. Kauppinen, S. Maruyama, *Adv. Energy Mater.***2017**, 7, 1700449.

[12] H. Wu, X. Zhao, Y. Sun, L. Yang, M. Zou, H. Zhang, Y. Wu, L. Dai, Y. Shang, A. Cao, *Solar RRL***2019**, 3, 1900147.

[13] Y. Qian, I. Jeon, Y. L. Ho, C. Lee, S. Jeong, C. Delacou, S. Seo, A. Anisimov, E. I. Kaupinnen, Y. Matsuo, Y. Kang, H. S. Lee, D. Kim, J. J. Delaunay, S. Maruyama, *Adv. Energy Mater.***2019**, 10, 1902389.

[14] D. D. Tune, N. Mallik, H. Fornasier, B. S. Flavel, *Adv. Energy Mater.***2019**, 10, 1903261.

[15] J. Chen, D. D. Tune, K. Ge, H. Li, B. S. Flavel, *Adv. Funct. Mater.***2020**, 30, 2000484.

[16] J. Chen, L. Wan, H. Li, J. Yan, J. Ma, B. Sun, F. Li, B. S. Flavel, *Adv. Funct.Mater.* **2020**, 30, 2004476.
